# Unprotected sexual practices and associated factors among adult people living with HIV on antiretroviral therapy in public hospitals of Kembata Tembaro Zone, Southern Ethiopia

**DOI:** 10.11604/pamj.2021.38.176.26105

**Published:** 2021-02-16

**Authors:** Dereje Laloto Anore, Bezatu Mengistie, Teketel Ermias Geltore

**Affiliations:** 1 Kembata Tembaro Zone Health Department, Durame, Ethiopia,; 2 School of Public Health, St. Paul Millennium Medical College, Addis Ababa, Ethiopia,; 3 Midwifery Department, School of Nursing and Midwifery, College of Health Sciences and Medicine, Wachemo University, Durame Campus, Durame, Ethiopia

**Keywords:** Unprotected sex, ART, HIV/AIDS, condom use, Ethiopia

## Abstract

**Introduction:**

Antiretroviral therapy (ART) significantly increases the life expectancy of HIV positive people by improving quality of life as well as enabling them to resume sexual activity. A growing number of people living with HIV became a source of exposure to sexually transmitted infections, including other strains of HIV that place others at risk unless they consistently use condoms. This study assessed the magnitude of unprotected sexual practices and associated factors among adult people living with HIV on ART in public hospitals of the Kembata Tembaro Zone, Southern Ethiopia.

**Methods:**

a facility-based cross-sectional study was conducted among adult people living with HIV on ART in public hospitals of the zone from March 1-30, 2016. Data were collected by a pretested and structured questionnaire. Binary logistic regression was used to investigate variables, independently associated with the outcome variable. The adjusted odds ratio with 95% CI used to show the strength of the association and a P-value < 0.05 was used to declare the cut-off point in determining the level of significance.

**Results:**

the study revealed that 40.9% of respondents practiced unprotected sexual intercourse. On multivariate logistic regression analyses, being females, having more than one sexual partner, mean monthly income of <530 Ethiopian birrs/ month, those who had a seropositive partner, a partner of unknown serostatus, and respondents who had insufficient knowledge of HIV transmission and prevention were statically significant with the outcome variable.

**Conclusion:**

the scarcity of knowledge on HIV transmission, negative attitude towards condom use, non-disclosure status and having more than one sexual partnership increased likelihood of have unprotected sex among the respondents.

## Introduction

Unprotected sex refers to sexual intercourse without male or female condom [1]. In sub-Saharan Africa, the most new Human immunodeficiency virus (HIV) infections were expected to occur in the heterosexual population of either in serodiscordant couples or because of having multiple sexual partners [2]. Globally, HIV virus continues to be a major public health issue, having claimed approximately 37.9 million people living with HIV, 1.7 million people became newly infected with HIV. About 32 million people had died from Acquired immune deficiency syndrome (AIDS)-related illnesses by the end of 2018 [3]. Sub-Saharan Africa is the most affected region with 25.8 million people living with HIV, and which accounts for almost 70% of global HIV infections [4].

According to an Ethiopian report, first in 1984, the HIV epidemic has evolved and then become a generalized epidemic. Now days, AIDS is the major cause of morbidity and mortality among adults in Ethiopia. In Ethiopia, there were an estimated 793,700 people living with HIV, including 200,300 children. There were approximately 45,200 AIDS- related deaths and about 898,400 AIDS orphans in 2013. HIV adult prevalence is 1.3% [5, 6]. The massive global expansion of Antiretroviral therapy (ART) access significantly improves physical, health and quality of life that enable individuals to resume sexual activity, including risky sexual practice [7, 8]. People living longer with HIV forms a potential source of infection with sexually transmitted infections; including other strains of HIV and places others at risk of HIV infection unless they practice safer sex [9]. Globally, unprotected sex among HIV positive people on ART became one of the problems that aggravated HIV transmission [10]. Having unprotected sex with regard to HIV infected person´s needs special attention because the risk of transmission to sero-discordant partners, and fear of infecting other HIV negative people. A study conducted in Africa reported that 43% of discordant rate among HIV positive people receiving ART [11, 12].

In addition, another fear of unprotected sex by HIV positive people on ART is the transmission of drug-resistant virus. The systematic review report of the different countries indicated the prevalence of transmitted drug resistance virus was 5.7% in Africa, 7.6% in Asia and 8.4% in Brazil [13]. The risk of transmission of drug-resistant virus to discordant, concordant and HIV serostatus negative people is evidenced by a significant number of HIV patients on ART engaging in unprotected sex. A study done in South Africa depicted that 34.0% having multiple sex partners and 24.2% having unprotected sex among people living with HIV on ART [14]. A study conducted in Kenya showed unprotected sex occurred in 52% of sexual partnerships with 32% of HIV-negative partners and 54% of partners of unknown HIV status in the last 6 months [2]. In Ethiopia, evidence indicates that significant numbers of HIV positive people on ART are practicing unprotected sex. A study conducted in Debra Zeit revealed that approximately 22.2% people living with HIV on ART practiced unprotected sex [15]. A similar study in Addis Ababa, showed the prevalence of unprotected sex among people living with HIV and using ART was 36.9% [8].

Southern Ethiopia is the third most populated region in Ethiopia with a total population of 14,929,548 according to the 2007 national census. The Ethiopian Demographic Health Survey of 2011 report indicated that the prevalence of HIV in the region was 1%, which showed the least prevalence in the country relative to other regions [16]. As report indicated that currently there are about 28,027 adults and 1671 pediatrics people living with HIV on ART, live in the South region. Despite, the number of HIV positive people on ART, there is little evidence about unprotected sexual practices of HIV positive people on ART in the study area. Therefore, this study was aimed to show the magnitude and factors associated with unprotected sexual practices among HIV positive people on ART, in public hospitals of Kembata Tembaro Zone (KTZ), Southern Ethiopia.

## Methods

### Study design

A facility-based cross sectional study design was employed using interviewer administered questionnaire. Additional information was obtained from ART registration books from March 1 to 30, 2016 in KTZ, Southern Ethiopia.

### Study setting and population

This study was conducted at Doctor Bogalech Gebre Memorial General Hospital and Shinshicho Primary Hospital of the Zone of Kembata Tembaro Zone. KTZ is located in Southern Ethiopia at 340 km from Addis Ababa, the capital city of Ethiopia. The estimated total population of the zone is 857,375 according to the 2008 zonal report. There are eight public health centers and two public hospitals providing ART services in this zone. Nine hundred and sixty-seven HIV positive people are on ART in the zone. Six hundred and six HIV positive people are on ART services in both Hospitals (204 in Shinshicho District Hospital and 402 in Doctor Bogalech Memorial General Hospital). According to KTZ Health Department, the total adult HIV positive population in both Hospitals was 535. The participants were sexually active adults attending an ART clinic/hospital of KTZ. Since the total study population in both Hospitals is manageable, all of the study populations available during the data collection period were included conveniently in the study.

### Data collection

Quantitative data collection tools were used to assess sexual behaviors among people on ART. Participants from ART clinics were interviewed using a pre-tested questionnaire translated to Amharic, the local language, with experts and back translation to English by another person to check the consistency. Two diploma-educated nurses were recruited and collected the data. Two public health officers´ supervised the data collectors with the permission and support of ART coordinators at the ART clinics. The questionnaire was intended to collect information on a socio-demographic characteristic of the respondents, perception-related questions, HIV status disclosure, childbearing and ART, sexual behaviors and ART, duration of access to ART, sexual behavior before HIV diagnosis. The interview was conducted as the eligible respondents are waiting to attend, or after they attended the service, at the ART clinics.

### Inclusion and exclusion criteria

The study included people receiving ARVs for at least 6 months and age 15 years and above (only the married if age below 18 years), who are healthy and not confined to bed. It is considered that patients on ART may receive different positive living messages, improvement of drug side effects, improvement of immunological response (increase Cluster of differentiation 4 (CD4+) count), and physical improvement and mental stability occur within3-6 months. Health care workers and peer educators who are on ART were excluded. In addition, those who could not speak, hear or were severely ill (those who cannot respond), and sexually inactive during 6 months prior to data collection were excluded.

### Assessment and definition of outcome and exposure variables

**Dependent:** unprotected sexual practices

**Independent:** socio- demographic, relationship factors and behavioral factors.

### Operational definitions

**Unprotected sexual practices:** defined as sexual intercourse without using a condom or inconsistent condom use with either HIV-negative, positive or unknown serostatus partners within six months before the study [17].

**Regular partner:** one with whom the respondent had a regular sexual relationship and who was perceived by the respondent to be the spouse or regular boy or girlfriend.

**Casual partner:** casual sex partner refers to the intention of one or both partners, usually the intention that this not turn into a relationship. One other than the regular partner with whom the respondent had sexual intercourse once or twice, or only having certain kinds of sex in the past six months with or without payment [18].

**Knowledge of HIV transmission:** knowledge of HIV/AIDS transmission means knowing at least two major HIV transmission prevention methods; abstinence and condom use and rejecting two major misconceptions, HIV transmission by mosquito bite and from kissing a person infected with HIV [19]. Those correctly answer all these questions were considered to have good knowledge about HIV transmission and those not correctly answering were considered to have poor knowledge. **Attitude to condom use:** an attitude is a relatively enduring organization of beliefs, feelings, and behavioral tendencies towards socially significant objects, groups, events or symbols [20]. In this study attitude of HIV positive, people on ART towards condom use during sexual intercourse. To determine the attitude of respondents towards condom use, respondents were asked attitude related questions on a five-point Likers scale ranged from ‘strongly disagree’ to ‘strongly agree’. The mean score of each respondent was dichotomized as having positive attitude or negative attitude.

### Data management and analysis

One-day orientation was given for data collectors and a supervisor on the use of the data collection tool. At the hospitals, data collectors were supervised by the supervisors and reported to the principal investigator daily. Before the actual data collection, a pretest was done with 5% of sample size at Shone hospital, which was outside of the study area and appropriate corrections were made before using it for the main study. Reliability of the tool was assessed with Cronbach´s alpha and found to be 0.84. Hosmer - Lemeshow goodness-of-fit statistic was done with p-value > 0.05 that indicated the model was good. After pre-testing, difficult questions were revised and adjusted, and then actual data collection was conducted. All completed questionnaires were checked for completeness and consistency, and double data entry was made using the Epidata 3.1 software. Data were then exported to SPSS version 16.0 for further analysis. Frequencies, proportions, and summary statistics were used to describe the study population in relation to relevant variables and presented in tables. The Chi-squared test was used to evaluate the association between current condom use levels and levels of condom use before testing positive HIV sero status. Bivariate analysis was carried out to identify variables that are significantly associated with risky sexual behavior. Those Variables in bivariate analysis whose p-value less than 0.25 were included in multivariate logistic regressions. Multivariate logistic regression analysis performed for those factors showed statistically significant in bivariate analysis to investigate independent predictors by controlling for possible confounders. Finally, the variables having an independent association with the practice of unprotected sexual intercourse were identified based on Adjusted odds ratio (AOR), with 95% CI and P-value < 0.05 were used to declare the cut-off point in determining the level of significance.

### Ethics approval and consent to participate

Ethical clearance was obtained from the ethical review board of the Institute of Health and Medical Sciences of Haramaya University. Permission was obtained from Shinshicho and Doctor Bogalech Gebre Memorial General Hospitals prior to the study. Written informed consent was obtained from study participants aged, 15 years and above (only the married if age below 18 years). For each person interviewed; the objectives, benefits of participating in the survey and progress of the investigation were clearly stated as well as their right to interrupt the interview without justification. Participant identification information was not collected to maintain anonymity and confidentiality. The interviews were conducted in a private room.

## Results

Of 535 questionnaires administered to respondents, 487 study subjects were interviewed and making response rate of 91%. Table 1 shows the socio-demographic characteristics of the participants. About 255 (52.4%) of the respondents were females. The mean age of respondents was 37.3 (SD+ 8.74). Most respondents, 408(83.8%) were Protestant Christians. Most respondents´ ethnicity was Kembata, which accounts for 380(78%). About 167(34.3%) were housewives.

**Table 1:** socio-demographic characteristics of the respondents among ART clients, Kembata Tembaro Zone public hospitals, 2016 (N=487)

Characteristics	Category	Frequency	Percent (%)
Sex	Male	232	47.6
	Female	255	52.4
Age in years	15-24	16	3.3
	25-34	181	37.2
	35-44	202	41.5
	45-54	74	15.2
	55-64	10	2.1
	Above 64	4	0.8
Marital status	Single	51	10.5
	Married	307	63
	Divorced	44	9
	Widowed/ Separated	85	17.4
Educational status	Illiterate	86	17.7
	1-8	273	56.1
	9-12	94	19.3
	Diploma	26	5.3
	Degree	8	1.6
Ethnicity	Kembata	380	78
	Hadya	53	10.9
	Wolayta	12	2.5
	Halaba	5	1.0
	Others*	37	7.6
Religion	Protestants	408	83.8
	Orthodox	42	8.3
	Muslim	19	3.9
	Catholic	10	2.1
	Others*	8	1.6
Income	>= 530 ETB	254	52.2
	< 530 ETB	233	47.8
Occupation	Student	5	1
	Farmer	120	24.6
	Housewife	167	34.3
	Gov´t employee	49	10.1
	NGO employed	8	1.6
	Self employed	101	20.7
	Others*	37	7.6

Ethnicity other*; other than the mentioned, Religion other* have no religion, Occupation Others* have no occupation

### Prevalence of unprotected sex among respondents on ART

**Figure 1:**
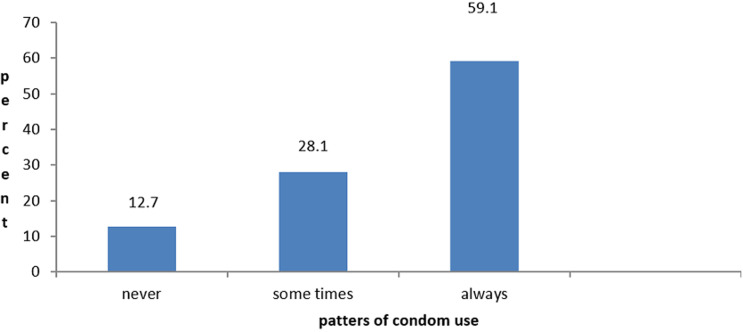
condom use practice of respondents 6 months prior to the study at Kembata Tembaro Zone public hospitals, 2016 (N=487)

Of 487 respondents who were sexually active adults attending ART clinic/hospital at the time of the data collection 6 months before the study, 199 (40.9%) practiced unprotected sex, of those 137 (28.1%) inconsistently use condom and 62 (12.8%) never used a condom. Figure 1 shows that 288 (59.1%) of respondents used condoms consistently. The result from the Chi-squared test, p = 0.15, on patterns of current condom use and condom use before HIV test positive, indicated that there is no evidence of differences in practiced unprotected sex between the two categories (condom use prior to 6 months vs. condom use before HIV test positive).

### Relationship factors

One hundred and twelve, (23%) of the respondents had engaged in sexual relations with more than one sexual partner within the six months before this study. 374 (77%) of reported having engaged in sexual intercourse with only one sexual partner of all types within the six months before the study. Most, 370 (76%) of the respondents currently had a regular partner currently 117 (24%) reported non-regular partner.

### Medical and behavioral factors associated with unprotected sex

Most 423(86.9%) of respondents on ART for more than 23 months and 13% were on ART for 6-23 months. One hundred and twelve (25.1%) reported that they had not disclosed their HIV status to their sexual partners and 365 (74.9%) respondents disclosed their HIV status to their sexual partners. Only 30 (6.2%) respondents had alcohol-consumed practices 6 months prior to the study. About 132 (27.1%) respondents reported that they desire to be bear children. Four hundred and fifty (92.4%) had sufficient knowledge and 37 (7.6%) had in sufficient knowledge of HIV transmission and prevention methods. Regarding the attitude of the respondents, most 278 (57.2%) had a positive attitude towards condom use and 208 (42.8%) respondents had a negative attitude. More than half, 267 (54.8%) of the respondents partners were HIV positive (Table 2).

**Table 2:** behavioral and medical factors associated with unprotected sex in Kembata Tembaro Zone public hospitals among ART clients, 2016 (N= 487)

Characteristics	Category	Frequency	%
HIV status of partner	HIV+	267	54.8
	HIV_	102	20.1
	Unknown	118	25.1
Alcohol consumption	Yes	30	6.2
	No	457	93.8
Desire of child	Yes	132	27.1
	No	355	72.9
Duration on ART ( in months)	6-23	64	13.1
	More than 23	423	86.9
Disclosed HIV status	Yes	370	74.9
	No	117	25.1
Knowledge on HIV transmission	Good knowledge	450	92.4
	Poor knowledge	37	7.6
Attitude on condom use	Positive attitude	278	57.2
	Negative attitude	208	42.8

### Reason for not using a condom

Multiple responses were possible, and the main reasons stated for not to using condom were; desire to have a child (66.3%), unavailability of condom during sexual intercourse (40.2%), partner refusal (22.6%), not thinking that condom use is important (12.5%), other reasons with very few responses included alcohol consumption during sexual intercourse and both partners being HIV positive.

### Factors associated with unprotected sex

Results of bivariate analysis showed that sex (p < 0.001), mean monthly income (p < 0.01), knowledge (P < 0.05), number of partners (p < 0.05), HIV status of partner (p < 0.01) and perception (p < 0.05) were significantly associated with unprotected sex (Table 3). Selected variables that were significantly associated with the bivariate analysis were further examined in logistic regression to see their relative effects in unprotected sexual practice. Sex, mean monthly income of the respondents, knowledge of HIV transmission, number of partners, type of partner, duration on ART and HIV status of partners were identified as independent predictors of unprotected sex. Females were 1.94 times more likely to practice unprotected sex compared with males (AOR: 1.94, 95% CI: 1.94(1.23, 3.08)). The odds of practicing unprotected sex were 2.3 fold higher in respondents who had more than one sexual partner compared with respondents who had only one sexual partner (AOR: 2.31, 95%CI: (1.33, 4.01)). Respondents who had non-regular (causal) partners were less likely to practice unprotected sex than those who had regular sexual partners (AOR: 0.28, 95%CI: (0.13, 0.62)). Respondents who had a mean monthly income of < 530 Ethiopian birrs per month were 1.8 times more likely to have unprotected sexual intercourse than respondents who had a mean monthly income of >= 530 Ethiopian birrs per month (AOR: 1.86, 95%CI: (1.21, 2.89)). Those who had a seropositive partner (AOR = 3.25, 95% CI: (1.76, 5.98)), were more likely to practice unprotected sex. Respondents who had insufficient knowledge of HIV transmission and prevention were 2.4 times more likely to had unprotected sex as those who had sufficient knowledge on HIV transmission and prevention (AOR: 2.37, 95%CI: (1.09,5.13)). Respondents who had survived for more than 23 months on ART were less likely to practiced unprotected sex than compared with respondents who survived on ART for 6-23 months(AOR = 0.42,95% CI: (0.23,0.79) (Table 4).

**Table 3:** factors associated with unprotected sex in bivariate analysis, Kembata Tembaro Zone public hospitals, 2016 (N=487)

Characteristics	Category	Unprotected sex						COR(95%CI)
		No	Frequency	%	Yes	Frequency	%	
Sex	Male		73	31.5		159	68.5	1
	Female		126	49.4		129	50.6	2.13(1.47,3.08**
Age	15-24		8	50		8	50	1
	25-34		81	44.8		100	55.2	0.81(0.29, 2.25)
	35-44		77	38.1		125	61.9	0.62(0.22, 1.71)
	45-54		28	37.8		46	62.2	0.61(0.21, 1.81)
	55-64		2	20		8	80	0.25(0.04, 1.55)
	Above 64		3	75		1	125	3.0(0.26,35.33)
Marital status	Single		125	49		26	51	1
	Married		115	37.5		192	62.5	0.62(0.34,1.13)
	Divorced		21	47.7		23	52.3	0.9(0.42,2.13)
	Widowed		38	47.5		47	62.5	0.84(0.42,1.69)
Number of partner	One		142	38		232	62	**1**
	More than one		57	50.9		55	49.1	1.69(1.11,2.59*
Residence	Urban		101	37.8		166	62.2	1
	Rural		98	44.5		122	55.5	1.32 (0.92,1.89)
Monthly income in ETB	>=530 ETB.		80	31.5		174	68.5	1
	<530 ETB.		119	51.1		114	48.9	2.27(1.57,3.28)**
HIV status of partner	HIV_ status		18	17.6		84	82.4	1
	HIV+ status		126	42.2		141	57.8	4.17(2.38,7.32)**
	unknown		55	46.6		63	53.4	4.07(2.18,7.61)**
Duration on ART	6-23 months		32	50		32	50	**1**
	More than 23 months		167	39.5		256	60.5	0.65(0.39, 1.11)
Disclosed HIV status	Yes		143	39.2		222	60.5	1
	No		56	45.9		66	54.1	1.32 (0.87,1.99)
Knowledge on HIV	Good knowledge		176	39.1		274	60.9	1
	Poor knowledge		23	62.2		14	37.8	2.56(1.28, 5.10)*
Attitude towards condom use	Positive attitude		106	38.1		172	61.9	1
	Negative attitude		92	44.2		116	55.8	1.29 (0.89, 1.85)

* P value <0.05, **P value < 0.001

**Table 4:** factors associated with unprotected sex in multivariate analysis, Kembata Tembaro Zone public hospitals, 2016 (N=487)

Characteristics	Category	Unprotected sex						AOR	P-value
		No	Frequency	%	Yes	Frequency	%	1	
Sex	Male		73	31.5		159	68.5	1.94(1.23,3.08)	0.001
	Female		126	49.4		129	50.6	**1**	
Number of partner	One		142	38		232	62	2.32(1.33,4.05)	0.05
	More than one		57	50.9		55	49.1		
Type of partner	Regular		151	40.8		219	59.2	1	
	Non-regular		48	41		69	59	0.28(0.13,0.61)	0.05
Monthly income in ETB	>=530 ETB.		80	31.5		174	60.5	**1**	
	<530 ETB.		119	51.1		114	48.9	1.87(1.21,2.89)	0.05
HIV status of partner	HIV_ status		18	17.6		84	82.4	1	
	HIV+ status		128	42.2		141	57.8	3.25(1.76,5.99)	0.001
	Unknown		55	46.6		63	53.4	5.04(.71,35.72)	0.106
Knowledge on HIV	Good knowledge		176	39.1		274	60.9	1	
	Poor knowledge		23	62.2		14	37.8	2.4(1.09,5.18)	0.05
Duration on ART	6-23 months		32	50		32	50	1	
	More than 23 months		167	39.5		256	60.5	0.42(0.23,0.75)	0.007

**Notes:** statistically significant in AOR: P-value <0.05

## Discussion

We showed that the prevalence of unprotected sex was (40.9%). Unprotected sex among females (49.4%) is higher than among males (31.5%). About (50%) of unprotected sex was seen in the age group of 15-24 years. Marital status of this study finding showed that single respondents practice unprotected sex more than married respondents. About 44.5% rural and 37.8% urbans residents practiced unprotected sex. Studies in different countries, revealed different levels of unprotected sex among HIV positive people on ART, ranging from 23.8% in Uganda to 51.2% in Nigeria respectively [21, 22]. Our study revealed that 40.9% of the respondents had unprotected (´risky´) sexual intercourse within the six months prior to the study. This is higher than reported in Uganda and Togo, where the prevalence of unprotected sex was 23.8%, 34.6% respectively [21, 23]. The reasons might be due to high reported desire to have a child, and the fact that most of the respondents were in a marital relationship in the present study. Another reason might be the difference in socio-demographic characteristics of study areas.

The prevalence of unprotected sex in the current study is lower than the reported in Nigeria, where the prevalence of unprotected sex was 51.2%, the reason might be the difference in socio-demographic characteristics of study areas [22]. In current study, regular partnership was the commonest partner type, and was 76%. This finding is similar to the study done in South Africa [14]. The present study revealed respondents reporting regular partnership were more likely to practice unprotected sex. The result of this study is consistent with the study conducted in Addis Ababa and South Africa [8, 14]. Unprotected sex occurred most often (57.3%) between sero-positive partners. The likelihood of unprotected sex was higher among those who knew their partner´s status to be HIV positive than those who did not know. This is which is similar with study findings from Nigeria and Uganda. On the other hand, this finding is lower than the study conducted in Ethiopia [8, 21, 22]. A point that should be critically noted here is that 8.2% and 25% of the unprotected sex was with partners perceived to be sero-negative or of unknown sero-status, respectively. This might contribute to new infection or re-infection with a new strain of HIV. Good numbers of partners were identified as an independent predictor of unprotected sex in the current study. Respondents who reported more than one partners were about two times more likely to have unprotected sex. This is consistent with the study done in Addis Ababa, Ethiopia [8].

There were significant differences in unprotected sex by gender. This study revealed that females were about two times more likely to practiced unprotected sex compared with males. It is comparable with the study findings from Nigeria. However, study findings from Addis Ababa, Ethiopia, reported that likelihood of unprotected sex being male practice was two times more likely compared to females. The reason for females more likely practice unprotected sex in this study might be their low-level income status. This is evidenced by most females being housewives in the current study [8, 22]. Mean monthly income was identified as an independent predictor of unprotected sex in this study. Respondents with low mean monthly income were two times more likely to practiced unprotected sex compared with those who reported high mean monthly income. This is similar to Uganda [21]. This study also identified participants´ knowledge of HIV transmission and prevention as independent predictor of unprotected sex. Respondents who had insufficient knowledge were two times more likely practiced unprotected sex. This is consistent with findings in Tanzania [24].

**Limitation of the study:** this is cross-sectional study, and the design does not determine the direction of causality. The sensitive nature of sexuality may result in social desirability bias, which may underestimate the prevalence of unprotected sex.

## Conclusion

More than one-third of respondents had unprotected sex in the six months prior to the study. The main reasons given for unprotected sex were partners´ dislike; both partners being HIV infected unavailability of condoms during sexual intercourse and the desire for a child. Being female having poor knowledge of HIV transmission, having negative attitude towards condom use, positive HIV status, low mean monthly income, and having more than one sexual partner increased likelihood of having unprotected sex. Ministry of Health and other stakeholders should be responsible for developing a guideline that gives greater attention how to address and should encourage safer sex among ART clients need to be developed and especial support may be needed to increase average monthly income status of ART patients. Health facilities managers should always check for accessibility and regular condom provision for ART client´s support and follow the comprehensive care provision at their facilities. Health care providers should; encourage free discussion among clients about condom use to enhance positive attitude towards condom use, create awareness on the risk of unprotected sexual intercourse and multiple sexual partner through regular counseling and health education and increase the knowledge of HIV positive people about HIV transmission and prevention methods.

### What is known about this topic

- Using ART is significantly increases the life expectancy of HIV positive people by improving physical, health and quality of life as well as enabling them to resume sexual activity;
- Practicing safer sex is important in preventing people living longer with HIV from a potential source of infection with sexually transmitted infections; including other strains of HIV and places others at risk of HIV infection;
- Using condoms regularly and consistently would protect the transmission of drug-resistant virus.

### What this study adds

- This is the first study to the area and identified the magnitude and factors associated with unprotected sexual practices among HIV positive people on ART;
- The study identified the extent of practice of unprotected sex in the six months prior to the study and factors contributing for not practicing. Considerations can be taken based on the factors identified for effective and consistent usage of condoms in study participants;
- The factors independently associated with unprotected sexual practices are the scarcity of knowledge on HIV transmission, negative attitude towards condom use, non-disclosure status and having more than one sexual partnership increased likelihood of have unprotected sex among the respondents.
